# Katatonie bei somatischer Erkrankung

**DOI:** 10.1007/s00115-024-01709-2

**Published:** 2024-07-25

**Authors:** Angela Luzia Herscheid, Nicoleta Carmen Cosma

**Affiliations:** 1 Klinik für Psychiatrie und Psychotherapie, Charité – Universitätsmedizin Berlin, corporate member of Freie Universität Berlin and Humboldt Universität zu Berlin, Hindenburgdamm 30, 12203 Berlin, Deutschland; 2 BIH Biomedical Innovation Academy, BIH Charité Clinician Scientist Program, Berlin Institute of Health at Charité – Universitätsmedizin Berlin, Berlin, Deutschland Charitéplatz 1, 10117

## Einführung

Selbst 150 Jahre nach Kahlbaums Erstbeschreibung „Die Katatonie: oder das Spannungsirresein“ bleibt die Katatonie unterdiagnostiziert [1]. Lange Zeit wurde die Katatonie als Unterform der Schizophrenie betrachtet. Die Katatonie als eigenständige Krankheitsentität in der 11. Version der Internationalen Klassifikation der Krankheiten (ICD-11) stellt einen willkommenen Anstoß dar, die Erkrankung erneut zu betrachten [2]. Das katatone Syndrom, bestehend aus gesteigerten, reduzierten und abnormen psychomotorischen Phänomenen, kann bei psychiatrischen Störungen, somatischen Erkrankungen oder substanzinduziert auftreten.

Zur Therapie der ersten Wahl stehen Benzodiazepine zur Verfügung. Beim Vorliegen maligner Symptome oder beim Nichtansprechen auf Benzodiazepine wird eine Elektrokonvulsionstherapie empfohlen [1]. Unter leitliniengerechter Behandlung wird für die Katatonie eine günstige Prognose beschrieben. Jedoch stellt die Katatonie, insbesondere in Assoziation mit somatischen Erkrankungen, ein unterdiagnostiziertes Krankheitsbild dar, welches unbehandelt mit einer erhöhten Morbidität und Mortalität einhergeht [3].

In der stationären psychiatrischen Versorgung wird die Katatonie mit einer Prävalenz von 5–18 %, in der neuropsychiatrischen Tertiärversorgung mit 3,3 % und 1,6–1,8 % im psychiatrischen Konsil- und Liaisondienstes (KL) beschrieben [3]. In der Literaturrecherche fällt auf, dass uns kaum Daten aus Deutschland vorliegen. Daher stellten wir uns die Frage, wie hoch die Prävalenz der Katatonie bei somatischer Erkrankung in einer deutschen Maximalversorgungsklinik ist, welche uns im Rahmen des KL präsentiert werden.

## Methodik

Für die Datenerhebung wurden alle vom 01.04.2022 bis zum 30.09.2023 an der Charité – Universitätsmedizin Campus Benjamin Franklin dem KL vorgestellten Fälle auf internistischen und chirurgischen Stationen verwendet. Zur retrospektiven Diagnosesicherung wurden die Kriterien der 5. Auflage des Diagnostischen und Statistischen Manuals Psychischer Störungen (DSM‑5, Katatonie aufgrund eines anderen medizinischen Krankheitsfaktors) und der ICD-11 (6E69) sowie das Bush-Francis Catatonia Screening Instrument (BFCSI) und die Rating Scale (BFCRS) retrospektiv angewandt. Wir prüften retrospektiv alle konsiliarischen Fragestellungen und Fälle individuell. Da in der ICD-11 und der DSM-5 ein zeitgleiches Vorliegen eines Delirs ein Ausschlusskriterium einer Katatonie darstellt, wurden die Fälle, bei denen ein Delir diagnostiziert wurde, ausgeschlossen [2, 4].

## Ergebnisse

In dem Zeitraum von 18 Monaten wurden insgesamt 1481 psychiatrische Konsile zu insgesamt 1008 Patient*innen auf somatischen Normal- und Intensivstationen bearbeitet. Fünf Katatoniefälle wurden identifiziert, welche zusammen zu 13 konsiliarischen Vorstellungen führten. Dies entspricht einer Prävalenz der Katatonie im KL von 0,50 %; von allen KL-Vorstellungen machen die Katatoniefälle 0,88 % aus. Alle Fälle wurden aus der neurologischen Abteilung vorgestellt (Tab. 1). Die Patient*innen hatten im Durchschnitt 3,8 positive Kriterien nach DSM‑5, 5,8 nach ICD-11 sowie durchschnittlich 6,2 Items des BFCSI erfüllt (Tab. 2). Während in 80 % eine Behandlung mit Benzodiazepinen durchgeführt wurde, wurde die Behandlung nur in einem Fall auf eine Elektrokonvulsionstherapie erweitert.

**Tab. 1:** Patient*innencharakteristika

*Demografie*		
Alter (Jahre) (M, SD)	60,6	21,77
Anzahl erfüllter DSM-5-Kriterien (von 12), (M, SD)	3,8	1,6
Anzahl erfüllter ICD-11-Kriterien (von 15), (M, SD)	5,8	1,83
Anzahl erfüllter Items BFCSI (von 14), (M, SD)	6,2	2,28
Anzahl erfüllter Items BFCRS (von 23), (M, SD)	6,4	2,06
Anzahl psychiatrischer Konsultationen, (M, SD)	2,6	1,36
Weiblich (*N*, %)	4	80
Kaukasisch (*N*, %)	5	100
Psychiatrische Vorerkrankungen (*N*, %)	3	60
Katatonie bei somatischer Erkrankung (*N*, %)	3	60
Katatonie bei psychiatrischer Erkrankung (*N*, %)	0	0
Katatonie nicht näher spezifiziert (*N*, %)	2	40
*Klinische Charakteristika (Aufnahmegrund)*	*Katatonierelevante Diagnosen*	
Fall 1, weiblich, 21 Jahre (Mutismus)	SARS-CoV-2-Infektion, Chromosom-18-Teildeletion	
Fall 2, weiblich, 72 Jahre (Vigilanzstörung)	Aspirationspneumonie, dekompensiertes Leberversagen	
Fall 3, weiblich, 58 Jahre (Vigilanzstörung, Tremor)	Lithiumintoxikation, Harnwegsinfekt, Folsäuremangel, paranoide Schizophrenie	
Fall 4, männlich, 79 Jahre (kognitive Störung)	SARS-CoV-2-Infektion, Z. n. kardialer Dekompensation und Urosepsis, schwerer depressiver Episode mit psychotischen Symptomen	
Fall 5, weiblich, 73 Jahre (Vigilanzstörung, Rigor)	Belastungsdyspnoe unklarer Ätiologie, Kachexie, rezidivierende depressive Störung, leichte kognitive Störung	

*M* Mittelwert, *N* Anzahl,* SD* Standardabweichung

**Tab. 2:** Verteilung katatoner Symptome nach DSM‑5, BFCSI, BFCRS, ICD-11 bei den Patient*innen

DSM‑5^a, e^	BFCSI^b^	BFCRS^c^	ICD-11^d, e^	Symptom zutreffend
–	–	–	*Reduzierte Psychomotorik:*	–
–	Staring	Staring	Staring	3
–	–	–	Ambitendenz	0
Negativismus	Negativismus	Negativismus	Negativismus	3
Stupor	Stupor	Stupor	Stupor	5
Mutismus	Mutismus	Mutismus	Mutismus	5
–	Rückzug	Rückzug	–	4
–	–	–	*Gesteigerte Psychomotorik:*	–
Agitation	Psychomotorische Erregung	–	Psychomotorische Erregung Extreme emotionale Reaktion Impulsivität Kampfbereitschaft	0 0 0 0
–	–	–	*Abnormale Psychomotorik:*	–
Grimassieren	Grimassieren	Grimassieren	Grimassieren	1
Manierismus	Manierismus	Manierismus	Manierismus	0
Posturing	–	–	Posturing	1
Stereotypie	Stereotypie	Stereotypie	Stereotypie	1
–	Rigor	Rigor	Rigor	4
Echolalie	Echophänomene	Echophänomene	Echophänomene	0
Echopraxie				
–	Verbigeration	Verbigeration	Verbigeration	0
Flexibilitas cerea	Flexibilitas cerea	Flexibilitas cerea	Flexibilitas cerea	1
Katalepsie	Posturing/Katalepsie	Posturing/Katalepsie	Katalepsie	4
–	–	Befehlsautomatie	–	1
–	–	Mitgehen	–	0
–	–	Gegenhalten	–	0
–	–	Perseveration	–	0
–	–	Autonome Dysregulation	–	2
–	–	Greifreflex	–	0

^a^Für die Diagnosestellung nach DSM‑5 müssen ≥ 3 der 12 Symptome erfüllt sein

^b^≥ 2 erfüllte Kriterien des BFCSI werden als positives Screening gewertet. Es wird empfohlen nur eindeutig positive Items zu zählen

^c^Für die BFCRS besteht kein einheitlicher Cut-off-Wert

^d^Für die Diagnosestellung nach ICD-11 müssen ≥ 3 Symptome aus einem oder mehrerer der Symptomcluster erfüllt sein

^e^Die Symptome werden nicht besser durch eine andere Erkrankung erklärt und führen zu einer relevanten Einschränkung

## Diskussion

Nur wenige Studien, größtenteils aus dem englischsprachigen Raum, berichten systematisch über die Katatonie bei somatischer Erkrankung. Dennoch ist die erschwerte Erkennung katatoner Syndrome im Allgemeinkrankenhaus gut belegt. Llesuy et al. zeigten, dass 59 % von Patienten mit Katatonie im somatischen Kontext nicht erkannt und somit nicht diagnostiziert wurden [3]. Die Anzahl der bei uns diagnostizierten Katatonien fällt im Vergleich zur englischsprachigen Literatur nochmals geringer aus.

In den vorliegenden englischsprachigen Studien wurde die Diagnose der Katatonie anhand des DSM oder mittels BFCSI gestellt. Auch wenn sich die Symptomkriterien dieser diagnostischen Ansätze überlappen, bestehen relevante Unterschiede. Für eine Katatonie nach DSM‑5 oder ICD-11 müssen jeweils 3 der entsprechenden Symptomkriterien erfüllt sein, nach BFCSI kann die Verdachtsdiagnose bereits bei 2 erfüllten Kriterien gestellt werden. Eine Untersuchung von Wilson et al. zeigte, dass nur 73 % der mittels BFCSI gestellten Verdachtsdiagnosen einer Katatonie tatsächlich die DSM-5-Kriterien erfüllten [5]. Darüber hinaus stellt das Delir nach DSM‑5 und ICD-11 eine Ausschlussdiagnose für eine Katatonie dar. Dies wurde in Studien basierend auf dem BFCSI nicht berücksichtigt [5]. Da sich die Symptome von katatonen und deliranten Syndromen überschneiden, könnte ein fehlender Ausschluss von Delirerkrankungen die jeweiligen Prävalenzen verfälschen. Außerdem könnte die geringe Assoziation zwischen motorischer Symptomatik und einer psychiatrischen Fragestellung zu einer verminderten psychiatrischen Konsilanfrage führen.

Ein einziges systematisches Review berichtete über 11 publizierte Fallserien von Katatonie im Allgemeinkrankenhaus. Dabei wurden 20 % der Katatoniefälle i. R. ausschließlich somatischer Erkrankungen, davon 77,9 % aufgrund einer ZNS-Erkrankung, berichtet [6]. In unserer Studie fällt ebenfalls auf, dass alle Katatoniefälle aus der neurologischen Abteilung stammen. Die Sensibilisierung für die Erkennung psychomotorischer Syndrome könnte dabei hilfreich sein.

Die ICD-11 weist erstmals eine Systematisierung in verminderte, gesteigerte und abnorme Psychomotorik auf, die sich im klinischen Alltag für die strukturierte Miterfassung katatoner Features im psychopathologischen Befund anbietet. Zusätzlich kann die Katatonie neu gesondert substanzinduziert diagnostiziert werden.

Zusammenfassend ist es unser Ziel mit dieser Arbeit auf das Krankheitsbild der Katatonie auf dem Boden somatischer Erkrankungen aufmerksam zu machen. Insbesondere wollen wir die Katatonie als eigenständige Krankheitsentität behandeln und somit dem inzwischen veralteten Bild der Katatonie, Erkrankungen aus dem schizophrenen Formenkreis vorenthalten zu sein, entgegenwirken.

## Fazit für die Praxis

- Die Katatoniefälle bei somatischer Erkrankung machen lediglich 0,50 % aller konsiliarisch vorgestellten Patient*innen in unserer untersuchten Stichprobe aus, deutlich niedriger als in der englischsprachigen Literatur vorbeschrieben.
- Die weiterhin verbreitete Assoziation mit schizophrenen Erkrankungen könnte zu einer Unterdiagnose der Katatonie führen.
- Die Einteilung in gesteigerte, verminderte und abnorme Psychomotorik in der ICD-11 könnte eine strukturiertere Erfassung der Katatonie ermöglichen.
